# What is the added value of FeNO as T2 biomarker?

**DOI:** 10.3389/falgy.2022.957106

**Published:** 2022-08-11

**Authors:** María Celeste Marcos, Carolina Cisneros Serrano

**Affiliations:** Department of Pulmonology, University Hospital La Princesa, Madrid, Spain

**Keywords:** asthma, biomarker, type 2 immune response, management, fraction of exhaled nitric oxide (FeNO)

## Abstract

There is increasing evidence about the role of nitric oxide in type 2 (T2) immune response. Fraction of exhaled nitric oxide (FeNO) is a product of airways inflammation and it is increased in patients with asthma. Since Gustaffson published the first article about this biomarker in the 1990s, interest has continued to grow. Compared with other T2 biomarkers such as blood eosinophil count, induced sputum, or serum periostin, FeNO has some remarkable advantages, including its not invasive nature, easy repeatability, and possibility to be performed even in patients with severe airway obstruction. It is considered as an indicator of T2 inflammation and, by the same token, a useful predictor for inhaled steroid response. It is difficult to determine the utility of nitric oxide (NO) for initial asthma diagnosis. In such a heterogenous disease, a single parameter would probably not be enough to provide a complete picture. There is also an important variability among authors concerning FeNO cutoff values and the percentage of sensibility and specificity for diagnosis. Its high specificity indicates a potential role to “rule in” asthma; however, its lower sensibility could suggest a lower capacity to “rule out” this pathology. For this reason, if a diagnosis of asthma is being considered, FeNO should be considered along with other tests. FeNO has also shown its utility to detect response to steroids, adherence to treatment, and risk of exacerbation. Even though there is not enough quality of evidence to establish overall conclusions, FeNO could be an alternative procedure to diagnose or exclude asthma and also a predictive tool in asthma treated with corticosteroids.

## Introduction

Asthma is a chronic inflammatory disease, characterized by reversible airway obstruction and bronchial hyperresponsiveness (1). However, in patients with mild asthma, airway obstruction is usually not present, which leads to diagnostic uncertainty. Thus, peak-flow measurement (low diagnostic value) or bronchial provocation (time-consuming, not always available, risk of bronchospasm) has been recommended for such situations (2). Therefore, in recent years, attention is more focused on a promising and simple tool (3).

Based on the degree and type of airway inflammation, a broad-spectrum of phenotypes has been identified (4), some of which have an eosinophilic inflammatory profile, while others may have increased levels of neutrophils. This inflammation, which is difficult to measure in clinical practice, is related to asthma symptoms and exacerbations.

Endogenous nitric oxide is derived from the inducible type of NO synthase (iNOS) present in airway cells including macrophages, neutrophils, epithelial, endothelial, and vascular smooth muscle cells. This enzyme utilizes L-arginine, a semi-essential amino acid, and oxygen as substrates (5). It is produced in response to airway inflammation, with iNOS expression induced by proinflammatory cytokines including tumor necrosis factor-α, interleukin-1b, and interleukin 4 (6, 7). Interleukin 13 (IL-13) may also upregulate the iNOS gene and therefore protein expression in epithelial cells (8)

NO has several roles in the regulation of pulmonary function. Thus, increased levels of FeNO in asthma are regarded as indirect markers of airway hyper-responsiveness, eosinophilic inflammation, and also associated with exacerbations and disease severity (9).

Measurement of FeNO concentration in exhaled air is a safe, economical, easy, and non-invasive method. Since Gustaffson (10) published the first article about this biomarker, several techniques have been developed. The chemiluminescence method is the gold standard for exhaled NO analysis (7). There is a highly standardized procedure for the measurement of FeNO. The patient makes an inhalation to total lung capacity and then exhales for 10 s at a specified pressure. According to international guidelines, it has to be done using a mean exhalation flow rate of 50 ml/s and instantaneous flows of 55 ml/s. In healthy adults, FeNO <25 parts per billion (ppb) is considered the normal value (11).

### FeNO as a biomarker of airway inflammation

A biomarker is a defined characteristic of a normal or pathogenic biologic process (12). It should be able to distinguish between health and disease, future risk, and response to treatment (13, 14).

Biomarkers such as FeNO, immunoglobulin E (IgE), or blood eosinophil counts are important tools to classify asthma patients into phenotypes. Correlation between biomarker levels and disease severity is limited. However, biomarker-based phenotyping (always associated with clinical evaluation) is important for an adequate characterization of patients and to define the best therapeutic strategy (14).

Although it generally correlates with eosinophilia, FeNO and eosinophilia are related to different type 2 cytokine pathways. Interleukin5 (IL-5) is involved in the activation, development, and recruitment of eosinophils, while Interleukin 4 (IL-4) and IL-13 are implied in IgE synthesis. They are distinct biomarkers for type 2 inflammation. This is the reason why FeNO should be considered a parallel marker of airway inflammation often, but not always, associated with eosinophilia (6). FeNO correlates with type 2 airways inflammation and has shown to be reduced with inhaled corticosteroids (ICS). Conversely, peripheral blood eosinophils originate from systemic circulation and its reduction is more significant with oral corticosteroid treatment (15). Simultaneously, increased blood eosinophil counts and FeNO could be associated with a higher prevalence of uncontrolled asthma: in an observational real-world cross-sectional study (16), patients with higher FeNO levels were characterized by lower body mass index, younger age, higher blood eosinophilia, atopy, higher airflow reversibility, more severe airflow obstruction, as well as persistent rhinitis and chronic rhinosinusitis with or without nasal polyps.

### Diagnostic accuracy

The American Thoracic Society (ATS) (11) recommends FeNO as part of the initial diagnosis of asthma. These guidelines define high FeNO levels in adults as >50 ppb, intermediate as 25–50 ppb, and low as <25 ppb. The current National Institute for Health and Care Excellence (NICE) guidelines (17) also propose the use of this tool for the initial diagnosis when asthma is suspected, with a cutoff value >40 ppb to support asthma diagnosis. GEMA (Guía Española para el Manejo del Asma) (4) suggests its utilization if the patient has asthma symptoms, spirometry is within normal limits, and the bronchodilator reversibility test is negative. Diagnosis is confirmed if the patient shows an adequate response to treatment. The BTS (British Thoracic Society) guidelines (18) consider that a positive FeNO test increases the probability of asthma, but a negative test does not exclude asthma. The global strategy for asthma management and prevention (GINA) guidelines (19) do not currently find a role for this biomarker in diagnostic algorithms. Recently, the European Respiratory Society (ERS) Guidelines for the Diagnosis of Asthma in Adults (20) also recommended measuring FeNO in patients suspected of asthma, when the diagnosis could not be established with initial spirometry and bronchodilator reversibility testing. Louis et al. propose a cutoff value of 40 ppb.

It is important to emphasize that regardless of FeNO level, pretest probability of asthma will influence posterior clinical decision-making. Likewise, in spite of pretest probability, a single FeNO measurement should not be interpreted alone (6, 15).

Utility of FeNO for asthma diagnosis is a controversial topic because of the disparity of the results in the accumulating evidence (21). There is an absence of evidence-based, patient-adjusted cutoffs, being one of the pending issues with FeNO measurement (6). Table 1 summarizes the diagnostic accuracy and cutoff values of FeNO.

**Table 1:** Diagnostic accuracy of FeNO in 5 meta-analysis with QUADAS-2 (22) quality assessment.

First author and year	Number of studies analyzed	Sensitivity (%)	Specificity (%)	Cutoff values
Li 2015 (23)	21	78%	74%	Cutoff values differed significantly (interstudy variation). Interstudy variation in asthma diagnosis standard
Guo 2016 (3)	25	72%	78%	Further studies are needed to verify optimal cutoffs for FeNO as a diagnostic aid in asthma
Harnan 2017 (24)	27	No sensitivity analyses were conducted		While optimal cutoff values failed to produce impressive accuracy, very high sensitivities and specificities were reported at low and high cutoffs, indicating the potential utility to rule in and/or rule out asthma
Schneider 2017 (1)	26	65%	82%	Optimal threshold around 60 ppb, and exclusion asthma might be possible with 20 ppb, when pretest probability of asthma is 30%
Karrasch 2017 (2)	26	65%	82%	A cut point around 50 ppb might guarantee a sufficient predictive positive value for ruling in asthma

### Predictive factor for exacerbations

Increased levels of FeNO are associated with a higher number of exacerbations. FeNO-guided management showed a significant reduction in exacerbations of any severity (25, 26). In a real-life study (27), FeNO demonstrated a stronger correlation with asthma exacerbations than peripheral blood eosinophils or periostin with no definite added benefit from a composite score of the three biomarkers. In a study by Busse and colleagues (28), 620 patients with uncontrolled, moderate-to-severe asthma were analyzed. Patients with baseline FeNO of 50 ppb or higher had a 1,54 times higher exacerbation rate than patients with FeNO of less than 25 ppb. Patients with baseline FeNO of 25 to <50 ppb had a 1.33 times higher exacerbation rate than patients with FeNO of less than 25 ppb. Furthermore, when combined with blood eosinophil count, the risk of exacerbation was higher in those with FeNO of 50 ppb or higher and blood eosinophil count of 150 cells per µl or higher (29). In asthmatics treated with inhaled corticosteroids, a low FeNO level could predict a low risk of exacerbation (30). McDowell (31) studied 145 patients with severe eosinophilic asthma in a multicenter observational cohort study. The participants were treated with mepolizumab and exacerbations were assessed. In the group with high sputum eosinophil count, exacerbations were FeNO high (>50 ppb) and with higher blood eosinophils count. On the other hand, the group with low sputum eosinophil count had higher C-reactive protein concentrations, lower FeNO (<20 ppb), and higher sputum neutrophils. FeNO was the most convenient discriminator of inflammatory phenotype at exacerbation.

The asthma guidelines of the Expert Panel Working Group of National Heart, Lung and Blood Institute (NHLBI) do not recommend the use of FeNO in isolation to predict or assess exacerbation frequency (32). This recommendation was likely directed at patients with mild-moderate asthma, in whom FeNO levels were not able to identify those at risk of severe exacerbations (29). Likewise, Pavord (33) studied the effects of as-needed budesonide-formoterol on exacerbations in patients with mild asthma and showed no evidence that FeNO was a prognostic biomarker for exacerbations

### Prediction of response to inhaled corticosteroids

In steroid-naïve asthma, a high FeNO level could predict a good response to ICS (30, 34).

After a randomized study conducted in 294 patients, Price (35) and cols concluded that treatment with ICS effects was effective for high FeNO: values higher than 50 ppb were associated with greater chances of improvement in cough on the visual analogue scale.

According to ATS guidelines (11), with FeNO values <25 ppb, eosinophilic inflammation and responsiveness to ICS measured by a post-bronchodilator forced expired volume in one second (FEV1) were unlikely, whereas with FeNO values >50 ppb, eosinophilic inflammation and responsiveness to ICS were likely.

Furthermore, low FeNO in asthmatics on regular ICS implies that increasing the glucocorticoid dose would not result in an improvement in the cure of asthma (36, 37).

On the other hand, a single-blind, parallel group, randomized controlled trial in adults using a composite score of T2 biomarkers (38) (including FENO) to adjust corticosteroid dose did not result in a greater proportion of patients reducing corticosteroid dose. FeNO should be used to help the addition of ICS to the treatment but not to suppress them.

However, there is a lack of good-quality data. The predictive ability of FeNO depends on the inflammatory phenotype of asthma; for this reason, the heterogeneity of asthma phenotypes in the mentioned studies could be a confounding factor, diminishing the real clinical benefit of FeNO measurements (30).

### Monitoring ICS adherence

Persistently high FeNO levels can be an indication of non-adherence. In a study of adult patients with poorly controlled asthma (39), FeNO suppression testing was an effective method to detect non-adherence to ICS. Following 1 week on ICS treatment, non-adherent patients experienced a substantial reduction in FeNO levels compared with adherent patients. ATS guidelines (11) suggest that FeNO could assist in the evaluation of adherence to anti-inflammatory medications. Also, NICE guidelines (40) indicate that FeNO is a useful marker for medicine adherence.

### Biological therapy

As FeNO can reflect the characteristics of the inflammatory profile and whether T2 inflammation may be present, it could be helpful in the selection of biologics for severe asthma (28, 41, 42).

In a large study of patients with severe asthma, Hanania (43) evaluated the benefit of omalizumab on exacerbations in relation to the presence of FeNO and peripheral blood eosinophils. If patients had elevated FeNO (>19,5% ppb), omalizumab reduced exacerbations by 53%, whereas the prevention of exacerbations was 19% when FeNO was <19,5% ppb. In patients treated with omalizumab, FENO ⩾19.5 ppb was related to longer time to first exacerbation, reduced exacerbation rate, and improvements in Asthma Quality of Life Questionnaire (AQLQ), compared with those with FENO <19.5 ppb. The ERS/ATS management of severe asthma guidelines (44) advocate using a FENO cutoff ⩾19.5 ppb to recognize patients with severe allergic asthma who are more likely to benefit from anti-IgE treatment.

Initial trials (45) with anti-IL-5 and anti-IL-5 receptor antibodies did not show FeNO to be a predictor of response. However, in a *post-hoc* analysis (46), patients with high baseline blood eosinophil levels experienced a greater reduction in exacerbations on mepolizumab treatment if they also had high baseline FeNO levels. Hoshino (47) combined FeNO and blood eosinophil count in 114 adult patients with type 2 asthma as a strategy to stratify them into 4 subgroups with distinct patterns of airway inflammation and of biologic use. The high-FeNO/high blood eosinophils and the high-FeNO/low blood eosinophils subgroups showed the largest prevalence of mepolizumab and benralizumab use. The high-FeNO/ low blood eosinophils had the largest frequency of acute exacerbations (AEs) and the shortest AE-free time in comparison with the other subgroups. This suggests that patients with severe asthma with solely high FeNO could be most likely to have refractory type 2 severe asthma.

Castro et al. (48) studied dupilumab in 1,900 patients (LIBERTY ASTHMA QUEST study). Significant dupilumab efficacy was observed in patients with FeNO⩾25 ppb or baseline levels of blood eosinophils ⩾150 μl^−1^. Nowadays, dupilumab is the only biologic drug with a therapeutic indication that other treatments do not have yet: FeNO levels are considered within the eligibility criteria. With regard to real-life studies about its effectiveness, Carpagnano et al. (49) demonstrated after 3 months of dupilumab biologic therapy an improvement in asthmatic symptoms (Asthma Control Test ACT pre 13.25 ± 4.65 vs ACT post 19.17 ± 4.45; *p* < 0.01), FeNO (32 ppb pre vs 19 pbb post) and lung function (FEV1% pre 62.58 ± 15.73 vs FEV1% post 71.00 ± 13.11; *p*< 0.01) in 12 severe type-2 asthma patients, a result that is in line with the multicenter, retrospective, real-world study published by Campisi et al (50). In this other study, patients were treated with dupilumab for 12 months. They observed an interruption of oral corticosteroids in all the patients, an increase in FEV1%, a significant decrease in the number of exacerbations, and an improvement in ACT.

A retrospective analysis of 162 subjects who attended the Difficult Asthma Centres in Belfast and Glasgow and experienced FeNO suppression testing (FeNOSuppT) was performed (51). This test consists in tele-monitoring of inhaler use in conjunction with daily FeNO measurement to identify non-adherence to inhaled corticosteroid (ICS) treatment. This study facilitates profiling for biomarker responses to corticosteroids to detect those patients more likely to respond to high-dose ICS therapy when used regularly and those who, despite good adherence, are likely to require additional treatment. This is the reason why FeNOSuppT could be useful in predicting progression to biologic agents.

Tezepelumab treatment (52, 53) showed a reduction in exacerbation rates and an improvement in lung function with a reduction in FeNO and blood eosinophils. The reduction in annual exacerbations with this biologic therapy was more pronounced with a high-baseline FeNO.

### Cost-effectiveness

FeNO is a cost-effective method. NICE guidelines (17, 40) concluded that incorporating a combination of peak expiratory flow measurement, spirometry bronchodilator reversibility test, FeNO, and methacholine challenge test was the most cost-effective strategy. Brooks and colleagues (54) reported that FeNO, in conjunction with standard of care guidelines for asthma management, decreased expected per-patient annual expenditure ($2,228) and increased expected per-patient annual quality-adjusted life year (QALY) (0.844) compared with standard of care (SOC) alone ($2,637 and 0.767). In Spain, according to Sabatelli (55), adding FeNO to standard asthma care saved €62,53 euros per patient and year and improved QALY.

### Limitations

– FeNO levels can also be increased in other conditions such as atopy and can also be affected by many other factors (for example, smoking reduces FeNO values). This is summarized in Table 2.
– FeNO is a marker of type 2 inflammation and therefore it plays less of a role in the diagnosis of non-type 2 asthma.
– Currently, GINA guidelines do not include the use of FeNO in their diagnostic pathway.
– There are still difficulties in establishing cutoffs for “high” and “low” values.
– FeNO testing is often unavailable in primary care, where most asthma diagnoses are made.

**Table 2:** Factors affecting FeNO levels.

Increased FeNO	Decreased FeNO
Atopy Atopic dermatitis Obesity Rhinovirus infection Eosinophilic bronchitis Allergic rhinitis COPD Lung transplant	Smoking Childhood

## Discussion

FeNO is a non-invasive, cheap, and non-time-consuming method. It provides an additional biomarker in the assessment of inflammatory airway diseases. It is accepted that increased FeNO levels are a surrogate of the T2 inflammatory pathway.

This technique can increase diagnostic accuracy in patients with asthma. High values of FeNO in patients with consistent symptoms confirm the suspicion of asthma.

It can be used to identify those patients who are more likely to respond to ICS. High FeNO values are associated with an increased probability of improvement with treatment.

Suppression of FeNO with ICS therapy allows a monitoring of adherence. Non-adherent patients tend to have higher FeNO levels despite the given treatment.

FeNO measurement can help in the phenotypization process of severe asthmatics to detect patients who may benefit from some of the biological therapies targeting type 2 inflammation. Moreover, patients with high levels of FeNO have a higher probability to respond to certain biologic treatments.

In conclusion, the use of FeNO should be encouraged as it provides an opportunity to increase diagnostic accuracy, optimize management, and tailor asthma treatment. There is a need for further research to establish adequate cutoff values and to define interaction with other biomarkers besides improving the access to this method.

## References

[1] Schneider A Linde K Reitsma JB Steinhauser S Rücker G. A novel statistical model for analyzing data of a systematic review generates optimal cutoff values for fractional exhaled nitric oxide for asthma diagnosis. J Clin Epidemiol. (2017) 92:69–78. 10.1016/j.jclinepi.2017.09.001 28916487

[2] Karrasch S Linde K Rücker G Sommer H Karsch-Völk M Kleijnen J Accuracy of FENO for diagnosing asthma: a systematic review. Thorax. (2017) 72(2):109–16. 10.1136/thoraxjnl-2016-208704 27388487

[3] Guo Z Wang Y Xing G Wang X. Diagnostic accuracy of fractional exhaled nitric oxide in asthma: a systematic review and meta-analysis of prospective studies. J Asthma. (2016) 53(4):404–12. 10.3109/02770903.2015.1101132 26796787

[4] Plaza V Alobid I Alvarez C Blanco M Ferreira J García G [Translated article] Spanish asthma management guidelines (GEMA) v.5.1. Highlights and controversies. Arch Bronconeumol. (2022) 58(2):T150–8. English, Spanish. 10.1016/j.arbres.2021.05.032 35971814

[5] Aytekin M Dweik RA. Nitric oxide and asthma severity: towards a better understanding of asthma phenotypes. Clin Exp Allergy. (2012) 42(5):614–6. 10.1111/j.1365-2222.2012.03976.x 22515385 PMC3335741

[6] Menzies-Gow A Mansur AH Brightling CE. Clinical utility of fractional exhaled nitric oxide in severe asthma management. Eur Respir J. (2020) 55(3):1901633. 10.1183/13993003.01633-2019 31949116

[7] Heffler E Carpagnano GE Favero E Guida G Maniscalco M Motta A Fractional Exhaled Nitric Oxide (FENO) in the management of asthma: a position paper of the Italian Respiratory Society (SIP/IRS) and Italian Society of Allergy, Asthma and Clinical Immunology (SIAAIC). Multidiscip Respir Med. (2020) 15(1):36. 10.4081/mrm.2020.36 32269772 PMC7137762

[8] Yamamoto M, Tochino Y, Chibana K, Trudeau JB, Holguin F, Wenzel SE. Nitric oxide and related enzymes in asthma: relation to severity, enzyme function and inflammation. Clin Exp Allergy. (2012) 42:760–8. 10.1111/j.1365-2222.2011.03860.x 22092728 PMC3650251

[9] Ricciardolo FL. Multiple roles of nitric oxide in the airways. Thorax. (2003) 58:175–82. 10.1136/thorax.58.2.175 12554905 PMC1746564

[10] Gustafsson LE Leone AM Persson MG Wiklund NP Moncada S. Endogenous nitric oxide is present in the exhaled air of rabbits, Guinea pigs and humans. Biochem Biophys Res Commun. (1991) 181(2):852–7. 10.1016/0006-291x(91)91268-h 1721811

[11] Dweik RA Boggs PB Erzurum SC Irvin CG Leigh MW Lundberg JO An official ATS clinical practice guideline: interpretation of exhaled nitric oxide levels (FENO) for clinical applications. Am J Respir Crit Care Med. (2011) 184(5):602–15. 10.1164/rccm.9120-11ST 21885636 PMC4408724

[12] Shute J. Biomarkers of asthma. Minerva Med. (2022) 113(1):63–78. 10.23736/S0026-4806.21.07381-X 34236156

[13] Tiotiu A. Biomarkers in asthma: state of the art. Asthma Res Pract. (2018) 4:10. 10.1186/s40733-018-0047-4 30598830 PMC6302401

[14] Carreiro-Martins P Regateiro FS Ferreira J Plácido JL Gerardo R Loureiro C. FeNO testing in severe asthma: a clinical argument or an access constraint? Pulmonology. (2021) 27(5):383–5. 10.1016/j.pulmoe.2021.05.002 34134929

[15] Loewenthal L Menzies-Gow A. FeNO in asthma. Semin Respir Crit Care Med. (2022). 10.1055/s-0042-1743290 [Epub ahead of print] 35253144

[16] Bertolini F Sprio AE Baroso A Riccardi E Pizzimenti S Carriero V Predictors of low and high exhaled nitric oxide values in asthma: a real-world study. Respiration. (2022) 101(8):746–56. 10.1159/000524498 35512642

[17] Asthma: diagnosis and monitoring of asthma in adults, children and young people. London: National Institute for Health and Care Excellence (NICE) (2017). PMID: 29206391. 29206391

[18] British Thoracic Society/Scottish Intercollegiate Guidelines Network. British guidelines on the management of asthma. A national guideline (2016).

[19] Global strategy for asthma management and prevention (2021 update). Global Initiative for Asthma (2021). https://ginasthma.org/reports/ (Accessed January 30, 2022).

[20] Louis R, Satia I, Ojanguren I, Schleich F, Bonini M, Tonia T, et al. European respiratory society guidelines for the diagnosis of asthma in adults. Eur Respir J. (2022):2101585. 10.1183/13993003.01585-2021 [Epub ahead of print] 35169025

[21] Ojanguren I Plaza V. FeNO for asthma diagnosis in adults: more lights than shadows. Arch Bronconeumol (Engl Ed). (2021) 57(2):85–6. English, Spanish. 10.1016/j.arbres.2020.03.035 32456799

[22] Whiting PF Rutjes AW Westwood ME Mallett S Deeks JJ Reitsma JB QUADAS-2: a revised tool for the quality assessment of diagnostic accuracy studies. Ann Intern Med. (2011) 155(8):529–36. 10.7326/0003-4819-155-8-201110180-00009 22007046

[23] Li Z Qin W Li L Wu Q Wang Y. Diagnostic accuracy of exhaled nitric oxide in asthma: a meta-analysis of 4,691 participants. Int J Clin Exp Med. (2015) 8(6):8516–24. 26309503 PMC4538098

[24] Harnan SE Essat M Gomersall T Tappenden P Pavord I Everard M Exhaled nitric oxide in the diagnosis of asthma in adults: a systematic review. Clin Exp Allergy. (2017) 47(3):410–29. 10.1111/cea.12867 27906490

[25] Essat M Harnan S Gomersall T Tappenden P Wong R Pavord I Fractional exhaled nitric oxide for the management of asthma in adults: a systematic review. Eur Respir J. (2016) 47(3):751–68. 10.1183/13993003.01882-2015 26846832 PMC4771622

[26] Petsky HL Kew KM Turner C Chang AB. Exhaled nitric oxide levels to guide treatment for adults with asthma. Cochrane Database Syst Rev. (2016) 9(9):CD011440. 10.1002/14651858.CD011440.pub2 27580628 PMC6457753

[27] Mansur AH Srivastava S Sahal A. Disconnect of type 2 biomarkers in severe asthma; dominated by FeNO as a predictor of exacerbations and periostin as predictor of reduced lung function. Respir Med. (2018) 143:31–8. 10.1016/j.rmed.2018.08.005 30261989

[28] Busse WW Wenzel SE Casale TB FitzGerald JM Rice MS Daizadeh N Baseline FeNO as a prognostic biomarker for subsequent severe asthma exacerbations in patients with uncontrolled, moderate-to-severe asthma receiving placebo in the LIBERTY ASTHMA QUEST study: a post-hoc analysis. Lancet Respir Med. (2021) 9(10):1165–73. 10.1016/S2213-2600(21)00124-7 34181876

[29] Chung KF. Increasing utility of FeNO as a biomarker of type-2 inflammation in severe asthma. Lancet Respir Med. (2021) 9(10):1083–4. 10.1016/S2213-2600(21)00170-3 34181878

[30] Lehtimäki L Csonka P Mäkinen E Isojärvi J Hovi SL Ahovuo-Saloranta A. Predictive value of exhaled nitric oxide in the management of asthma: a systematic review. Eur Respir J. (2016) 48(3):706–14. 10.1183/13993003.00699-2016 27492830

[31] McDowell PJ Diver S Yang F Borg C Busby J Brown V The inflammatory profile of exacerbations in patients with severe refractory eosinophilic asthma receiving mepolizumab (the MEX study): a prospective observational study. Lancet Respir Med. (2021) 9(10):1174–84. 10.1016/S2213-2600(21)00004-7 33971168

[32] Expert Panel Working Group of the National Heart, Lung, and Blood Institute (NHLBI) administered and coordinated National Asthma Education and Prevention Program Coordinating Committee (NAEPPCC), Cloutier MM Baptist AP Blake KV Brooks EG Bryant-Stephens T 2020 Focused updates to the asthma management guidelines: a report from the national asthma education and prevention program coordinating committee expert panel working group. J Allergy Clin Immunol. (2020) 146(6):1217–70. 10.1016/j.jaci.2020.10.003. Erratum in: *J Allergy Clin Immunol*. (2021) 147(4):1528-1530 33280709 PMC7924476

[33] Pavord ID Holliday M Reddel HK Braithwaite I Ebmeier S Hancox RJ Predictive value of blood eosinophils and exhaled nitric oxide in adults with mild asthma: a prespecified subgroup analysis of an open-label, parallel-group, randomised controlled trial. Lancet Respir Med. (2020) 8(7):671–80. 10.1016/S2213-2600(20)30053-9 32171064

[34] Szefler SJ Martin RJ King TS Boushey HA Cherniack RM Chinchilli VM Significant variability in response to inhaled corticosteroids for persistent asthma. J Allergy Clin Immunol. (2002) 109(3):410–8. 10.1067/mai.2002.122635 11897984

[35] Price DB Buhl R Chan A Freeman D Gardener E Godley C Fractional exhaled nitric oxide as a predictor of response to inhaled corticosteroids in patients with non-specific respiratory symptoms and insignificant bronchodilator reversibility: a randomised controlled trial. Lancet Respir Med. (2018) 6(1):29–39. 10.1016/S2213-2600(17)30424-1 29108938

[36] Kupczyk M Haque S Middelveld RJ Dahlén B, Dahlén SE; BIOAIR investigators. Phenotypic predictors of response to oral glucocorticosteroids in severe asthma. Respir Med. (2013) 107(10):1521–30. 10.1016/j.rmed.2013.07.014 23993706

[37] Michils A Baldassarre S Van Muylem A. Exhaled nitric oxide and asthma control: a longitudinal study in unselected patients. Eur Respir J. (2008) 31(3):539–46. 10.1183/09031936.00020407 18057062

[38] Heaney LG Busby J Hanratty CE Djukanovic R Woodcock A Walker SM Composite type-2 biomarker strategy versus a symptom-risk-based algorithm to adjust corticosteroid dose in patients with severe asthma: a multicentre, single-blind, parallel group, randomised controlled trial. Lancet Respir Med. (2021) 9(1):57–68. 10.1016/S2213-2600(20)30397-0. Erratum in: Lancet Respir Med. 2021 Jan 4. 32916135 PMC7783382

[39] Heaney LG Busby J Bradding P Chaudhuri R Mansur AH Niven R Remotely monitored therapy and nitric oxide suppression identifies nonadherence in severe asthma. Am J Respir Crit Care Med. (2019) 199(4):454–64. 10.1164/rccm.201806-1182OC 30339770

[40] Akehurst R, Cole T, Collinson P, Crawford S, Cree I, Denton E, et al. National Institute for Health and Care Excellence (NICE). Diagnostics guidance. Measuring fractional exhaled nitric oxide concentration in asthma: NIOX MINO, NIOX VERO and NObreath (2014). www.nice.org.uk/guidance/dg12.

[41] Lombardi C Berti A Cottini M. The emerging roles of eosinophils: implications for the targeted treatment of eosinophilic-associated inflammatory conditions. Curr Res Immunol. (2022) 3:42–53. 10.1016/j.crimmu.2022.03.002 35496822 PMC9040157

[42] Busse WW. Biological treatments for severe asthma: a major advance in asthma care. Allergol Int. (2019) 68(2):158–66. 10.1016/j.alit.2019.01.004 30792118

[43] Hanania NA, Wenzel S, Rosén K, Hsieh HJ, Mosesova S, Choy DF, et al. Exploring the effects of omalizumab in allergic asthma: an analysis of biomarkers in the EXTRA study. Am J Respir Crit Care Med. (2013) 187:804–11. 10.1164/rccm.201208-1414OC 23471469

[44] Holguin F Cardet JC Chung KF Diver S Ferreira DS Fitzpatrick A Management of severe asthma: a European Respiratory Society/American Thoracic Society guideline. Eur Respir J. (2020) 55(1):1900588. 10.1183/13993003.00588-2019 31558662

[45] Pavord ID Korn S Howarth P Bleecker ER Buhl R Keene ON Mepolizumab for severe eosinophilic asthma (DREAM): a multicentre, double-blind, placebo-controlled trial. Lancet. (2012) 380(9842):651–9. 10.1016/S0140-6736(12)60988-X 22901886

[46] Shrimanker R Keene O Hynes G Wenzel S Yancey S Pavord ID. Prognostic and predictive value of blood eosinophil count, fractional exhaled nitric oxide, and their combination in severe asthma: a *Post Hoc* analysis. Am J Respir Crit Care Med. (2019) 200(10):1308–12. 10.1164/rccm.201903-0599LE 31298922

[47] Hoshino Y Soma T Uchida Y Shiko Y Nakagome K Nagata M. Treatment resistance in severe asthma patients with a combination of high fraction of exhaled nitric oxide and low blood eosinophil counts. Front Pharmacol. (2022) 13:836635. 10.3389/fphar.2022.836635 35517829 PMC9065285

[48] Castro M, Corren J, Pavord ID, Maspero J, Wenzel S, Rabe K, et al. Dupilumab efficacy and safety in moderate-to-severe uncontrolled asthma. N Engl J Med. (2018) 378:2486–96. 10.1056/NEJMoa1804092 29782217

[49] Carpagnano GE Scioscia G Buonamico E Lacedonia D Diaferia F Capozza E Early effectiveness of type-2 severe asthma treatment with dupilumab in a real-life setting; a FeNO-driven choice that leads to winning management. Multidiscip Respir Med. (2022) 17(1):797. 10.4081/mrm.2022.797 35280851 PMC8848342

[50] Campisi R Crimi C Nolasco S Beghè B Antonicelli L Guarnieri G Real-World experience with dupilumab in severe asthma: one-year data from an Italian named patient program. J Asthma Allergy. (2021) 14:575–83. 10.2147/JAA.S312123 34079295 PMC8167193

[51] Butler CA McMichael AJ Honeyford K Wright L Logan J Holmes J Utility of fractional exhaled nitric oxide suppression as a prediction tool for progression to biologic therapy. ERJ Open Res. (2021) 7(3):00273–2021. 10.1183/23120541.00273-2021 34549044 PMC8450452

[52] Menzies-Gow A Corren J Bourdin A Chupp G Israel E Wechsler ME Tezepelumab in adults and adolescents with severe, uncontrolled asthma. N Engl J Med. (2021) 384(19):1800–9. 10.1056/NEJMoa2034975 33979488

[53] Corren J Parnes JR Wang L Mo M Roseti SL Griffiths JM Tezepelumab in adults with uncontrolled asthma. N Engl J Med. (2017) 377(10):936–46. 10.1056/NEJMoa1704064. Erratum in: N Engl J Med. 2019 May 23;380(21):2082. 28877011

[54] Brooks EA Massanari M. Cost-Effectiveness analysis of monitoring fractional exhaled nitric oxide (FeNO) in the management of asthma. Manag Care. (2018) 27(7):42–8. PMID: 29989901

[55] Sabatelli L Seppälä U Sastre J Crater G. Cost-effectiveness and budget impact of routine use of fractional exhaled nitric oxide monitoring for the management of adult asthma patients in Spain. J Investig Allergol Clin Immunol. (2017) 27(2):89–97. 10.18176/jiaci.0103 27564130

